# Health-Related Quality of Life and Function after Paediatric Injuries in India: A Longitudinal Study

**DOI:** 10.3390/ijerph14101144

**Published:** 2017-09-28

**Authors:** Jagnoor Jagnoor, Shankar Prinja, Aliki Christou, Jannah Baker, Belinda Gabbe, Rebecca Ivers

**Affiliations:** 1 The George Institute for Global Health, University of New South Wales (UNSW), Sydney 2052, Australia; achristou@georgeinstitute.org.au (A.C.); jbaker1@georgeinstitute.org.au (J.B.); rivers@georgeinstitute.org.au (R.I.); 2 School of Public Health, Post Graduate Institute for Medical Education and Research, Chandigarh 160012, India; shankarprinja@gmail.com; 3 Sydney School of Public Health, The University of Sydney, Sydney 2006, Australia; 4 School of Public Health and Preventive Medicine, Monash University, Melbourne 3004, Australia; belinda.gabbe@monash.edu; 5 Farr Institute, Swansea University Medical School, Swansea University, Swansea SA2 8PP, UK

**Keywords:** paediatric, trauma, quality of life

## Abstract

Paediatric injuries can lead to long-term functional impairment and reduced health-related quality of life, and are a growing public health issue in India. To date, however, the burden has been poorly characterized. This study assessed the impact of non-fatal injuries on health-related quality of life in a prospective cohort study of 373 children admitted to three hospitals in Chandigarh and Haryana states in India. The Pediatric Quality of Life Inventory (PedsQL) and King’s Outcome Scale for Childhood Head Injury (KOSCHI) were administered at baseline (pre-injury) and at 1, 2, 4, and 12 months post-injury by telephone interview. Follow-up at all-time points was completed for 277 (77%) of all living participants. Less than one percent reported ongoing disability at 4 months, and no disability was reported at 12 months. PedsQL physical health scores were below healthy child norms (83.4) at 1 month in the cohort for ages 8–12 years and 13–16 years. Although injuries are prevalent, ongoing impact on functioning and disability from most childhood injuries at 12 months was reported to be low. The results raise questions about reliability of generic, Western-centric tools in low- and middle-income settings, and highlight the need for local context-specific tools.

## 1. Introduction

The burden of unintentional injuries falls disproportionately on children, as highlighted in the 2008 World Report on Child Injury [1] and recent global burden of disease estimates which found that injuries, particularly road traffic injuries, were the leading cause of death among 10–14 year olds [2]. Even more substantial but less well documented are non-fatal injuries which lead to hospitalisation, disability, and other long-term consequences in functioning and quality of life. In 2010, injuries caused 11% of disability adjusted life years (DALYs) [3] and more recent global estimates suggest that unintentional injuries are the second leading cause of years lived with disability among 10–24 year olds [4]. The impact of such injuries can have not only physical, but psychosocial and economic consequences for the injured person and their family. In India, studies have also demonstrated disproportionate impact of injuries on children, who comprise over one-third of injury cases in rural community settings [5] and urban hospital settings [6]. However, the impact of injuries on health-related quality of life, specifically on functioning for children, has not been explored in many low- and middle-income countries. This prevents true understanding of the actual cost to families and communities and inhibits investment in both prevention and appropriate clinical care.

Paediatric injuries represent an important subset of the trauma population, with lower mortality rates than adult populations, but with likelihood of lifelong functional impairment and disability. Children and adults differ in their interpretation and reporting of health outcomes, requiring paediatric-specific instruments for measuring outcomes. Research in the past has highlighted the importance of capturing functional and health-related quality of life (HRQoL) data after paediatric health conditions, including the challenges of collecting this information [7,8].

Focusing on mortality data or discharge disposition data only may result in strategies ignoring post-discharge recovery; longer follow-up is essential to understanding the true impact. The aim of this study was to assess the short- and longer-term outcomes of injured children requiring hospitalisation in an urban setting in India, using a selection of functional and HRQoL instruments, informing on non- fatal injury burden in children. 

## 2. Materials and Methods

### 2.1. Study Design and Setting

The study protocol has been previously published and was extended to include all injuries, at all ages [9]. Patients were recruited from three health care facilities: the Postgraduate Institute of Medical Education and Research (PGIMER), a tertiary care institution; the Government Multi-Speciality Hospital (GMSH), a secondary care institution; and Government Hospital, a secondary care institution. Selection of the hospitals was purposive, as these catered to large volumes of trauma patients. 

The Postgraduate Institute of Medical Education and Research (PGIMER) is a large 1850 bedded super specialty hospital. Advanced Trauma Centre (ATC) [10] of the institute is a specialized and fully equipped centre catering to the needs of trauma victims. In addition to the injury patients who come to ATC directly, this hospital serves as a referral centre for hospitals in the city and neighbouring states including Punjab, Haryana, Himachal Pradesh, and Uttar Pradesh. The Government Multi-Speciality Hospital (GMSH) is a 500 bedded secondary care hospital providing care to the residents of Chandigarh and the surrounding states. The District level hospital is a 300 bedded secondary care hospital located in Panchkula district of Haryana. Trauma patients are treated along with other emergency patients at the emergency centre of the hospital. Severe trauma injury patients in this hospital are referred to other tertiary care hospitals in the region. A total of 11,511 trauma patients received care at this facility during March 2014–March 2015.

### 2.2. Study Participants and Eligibility Criteria

Children aged 2–16 years were eligible for inclusion in this analysis from the prospective cohort study. Inclusion criteria were broad, with an overnight admission at the study hospitals due to any injury as defined by Internal Classification of Disease (ICD) 10 codes [11] for external causes of morbidity and mortality resulting from road crashes, falls, burns, mechanical injuries, animal bites, poisoning, drowning, etc. In total, 387 children were enrolled in the study over the period from April 2015 to August 2015 (inclusive). 

### 2.3. Recruitment Procedures and Follow-Up

Participants were recruited during their inpatient stay at the three study hospitals. Records were screened for all admitted patients with an injury and other eligibility criteria. Informed consent was obtained from parents of the child, and consent from the child was obtained for those aged 7–17 years, as advised by the Indian Council of Medial Research ethical guidelines. The ethical review committee of Postgraduate Institute for Medical Education and Research and institutional review boards of each participating hospital approved the study (Approval No. PGI/IEC/2014/2262).

The patient’s identified primary caregiver (child’s mother in 87% of cases) completed the surveys including the HRQoL at all four time points. In the case of the primary caregiver’s absence, another caregiver capable of reporting accurate responses completed the survey. Baseline interviews were administered face-to-face at the hospital by trained staff. Follow-up interviews were completed at 1, 2, 4, and 12 months post-injury.

### 2.4. Measures

Parents completed a questionnaire at baseline which captured demographic information, socio-economic status, and circumstances around injury and hospitalisation. The mechanism of injury was defined in accordance with Internal Classification of Disease (ICD 10), Chapter XX—External causes of morbidity and mortality [12]. Place and activity during injury were also recorded as per ICD 10. Comorbidities were defined as any medical condition requiring medical attention, as an open text. Socio-demographic variables were based on Census, India classification.

### 2.5. The King’s Outcome Scale for Childhood Head Injury

The King’s Outcome Score for Child Head Injury (KOSCHI) is a paediatric adaptation of the extended (eight-level) adult Glasgow Outcome Scale (GOS) [13]. The eight categories are as follows: death, vegetative state, lower severe disability, upper severe disability, lower moderate disability, upper moderate disability, good recovery, and intact recovery. The KOSCHI was administered for all children in the study.

### 2.6. The Paediatric Quality of Life Inventory

Health-related quality of life was assessed using the Pediatric Quality of Life Inventory Generic Core Scales (PedsQL). The Pediatric Quality of Life Inventory (PedsQL, V4) is a 23-item generic HRQL instrument designed to measure the four dimensions of physical, mental, social health, and school functioning. The instrument uses a five-point Likert response scale to assess the extent to which different items have affected the child in the previous 4 weeks. The recall period is 1 month and items have five response options ranging from 0 ‘never’, to 4 ‘almost always a problem’. Individual item scores are obtained by reverse scoring items and linearly transforming them to a scale of 0 to 100, with 100 representing perfect health. Total scores are obtained by adding the sum of items and dividing them by the number of items answered. Responses to each of the items are used to generate physical health and psychosocial health summary scores. The summary scores range from 0 to 100, with higher scores representing better function. The parent report version was used for 2–7 years, child report for 8–12, and teen age reports for 13–16 teen years. 

The instruments utilised were chosen based on their ability to measure important dimensions of quality of life, functioning, and disability [8,14,15] as defined by the International Classification of Functioning, Disability and Health (ICF), as well as brevity, and the capacity to be administered by telephone interview. They are also applicable to a broad range of injuries and age groups, and have been recommended as being valid and appropriate measures of the impact of injury in high-income context, and validity in low and middle income country (LMIC) for injuries is ambiguous. Instruments, using validated translated Hindi versions, were used in this study [16].

### 2.7. Statistical Analysis

Statistical analyses were carried out in SAS 9.4 with SAS/STAT 14.2 (SAS Institute, Cary, NC, USA).

PedsQL as the main outcome measure. Each PedsQL item was reverse scored and linearly transformed to a scale of 0 to 100; therefore, higher scores reflected better HRQoL. For each caregiver or patient respondent, a total, physical, or psychosocial summary score was computed as the sum of the items divided by the number of items answered. The psychosocial score was composed of the emotional, social, and school functioning scores. The KOSCHI scores were ranked in order of severity: 1 = 1, 2 = 2, 3a = 3, 3b = 4, 4a = 5, 4b = 6, 5a = 7, 5b = 8.

## 3. Results

**Figure 1:**
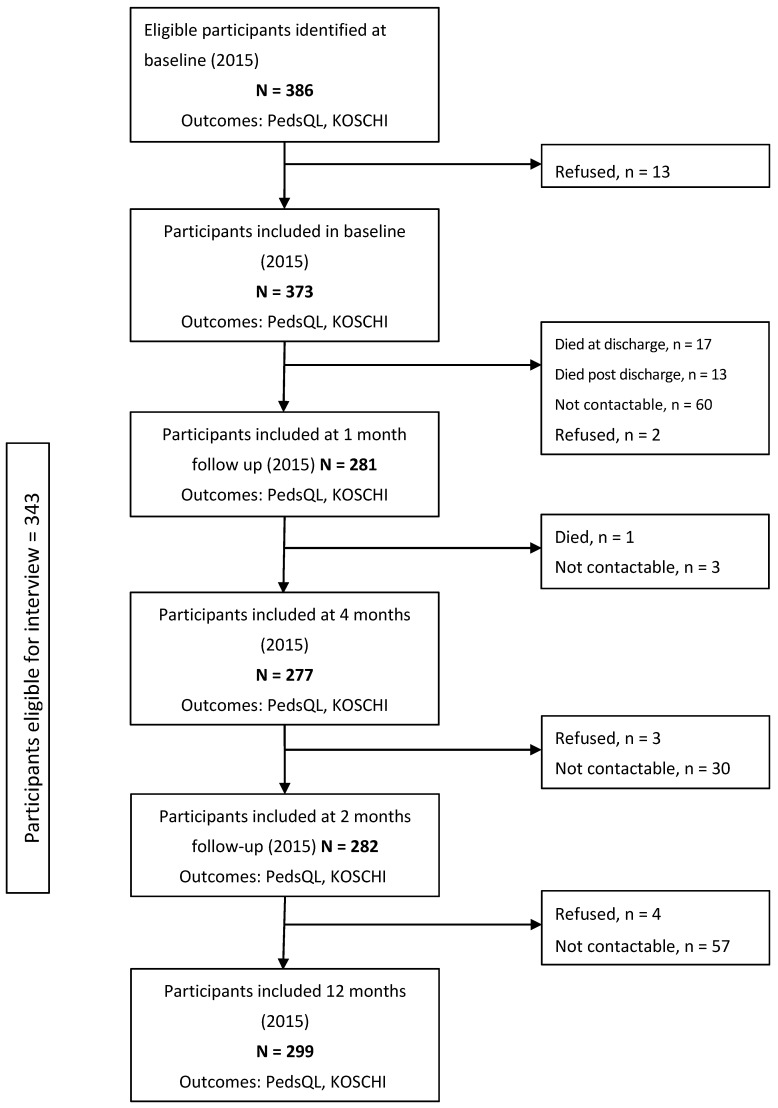
Flow chart of prospective cohort study. PedsQL: Pediatric Quality of Life Inventory; KOSCHI: King’s Outcome Scale for Childhood Head Injury.

Out of the 386 eligible injured children identified, 373 were included in the study at baseline. Of those, 281 were followed up at one month while follow-up at all-time points was completed for 72% of cases (*n* = 277/386) (Figure 1). 

Demographic and injury characteristics of participants are shown in Table 1. Participants were equally distributed across all age groups, and almost three-quarters of participants were male. The majority of those aged over 6 years had primary level education, while around a quarter had no education. Almost all injuries were the result of unintentional injury events. Falls were the predominant mechanism of injury, with almost half of participants being admitted as result of falls, followed by road traffic injuries, which accounted for over a third of injuries. Mechanical injuries and burns were the third and fourth most common injury mechanisms. About half of injuries occurred at home or at a residential institution, and about one-third on a street or highway. A third of participants reported a mild injury severity score, half reported moderate, and 15% reported severe. Surgery was required in less than 2% of injury cases. 

Table 2 shows specific details for road traffic injuries (RTIs) only. One-third of injuries were sustained by pedestrians and those driving two-wheeler vehicles. In almost half the cases, the passenger was the injured person. For injuries involving two-wheeler vehicles, helmet wearing was reported by only 11% of injury cases. For injuries sustained in a car, seatbelt or use of child restraint was not reported in any of the cases.

Fall injuries are summarized in Table 3. Over one-third (37%) of fall injuries occurred from a roof or building, while 15% were sustained while climbing on stairs/steps or ladder. Over a third of cases did not specify the type of fall that caused the injury. In the majority of cases, a slippery surface was cited as the reason for the fall (Table 2).

### Functional and Health-Related Quality of Life Outcomes

**Figure 2:**
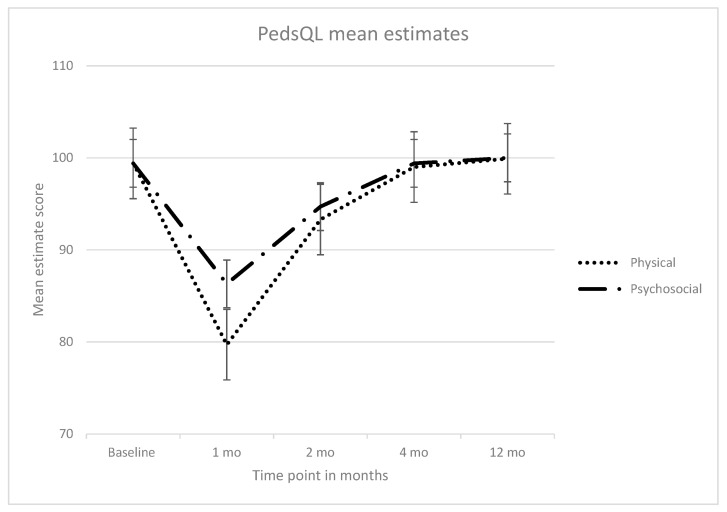
PedsQL mean estimates for children aged 2–16 years at baseline, 1, 2, 4, and 12 months after injury (*n* = 386); *p* < 0.007 comparison to population norms.

Functional and health-related quality of life outcomes of children at each of the four time points are summarized in Table 4 and Table 5, and Figure 2. At baseline, the mean physical and psychosocial PedsQL scores among all children was 99.4 (SD: 3.4). This declined after one month to 79.7 for physical wellbeing and to 86.3 for the psychosocial wellbeing, however, all scores improved after the two month follow-up time point. Improvement in psychosocial wellness occurred at a greater rate than physical wellness.

Table 5 shows the PedsQL scores by age group at each time point. Children in the older age groups had lower physical scores than the younger age groups at one month follow-up, by two months follow-up these differences were marginal.

## 4. Discussion

This study assessed outcomes of children following a traumatic injury using functional and HRQoL instruments. To our knowledge, this is the first research study reporting on HRQoL after trauma in India and application of these instruments in this setting.

In this study, falls were the predominant cause of injury admissions among children at the three hospitals included, with most of these falls occurring in the home. Injury prevention efforts in low-income countries have focused heavily on road traffic injuries, with falls receiving very little attention. Falls are also known to be a major cause of child injury deaths in India [17]. Falls from the roof were common and mostly associated with a slippery surface. Road traffic injuries were the second leading cause with pedestrians, bi-cyclists, and two-wheeler occupants at highest risk, and very low helmet wearing rates reported. 

Overall, there was evidence of rapid improvement in function and HRQoL after one month in this cohort of Indian children and there did not appear to be any ongoing or long-term functional loss or reduction of HRQoL at 12 months post-injury, suggesting good recovery. While there are no studies in LMIC settings to compare to, the recovery outcomes appear to be better for those in the moderate to severe injury cohort than previously reported in high-income country settings using the same instruments. Research from high-income contexts has also reported that regardless of injury severity and the instrument used to measure HRQoL, most injured children recovered quickly, and had regained baseline HRQoL status by four months post-injury [15,18,19]. 

A validation study amongst adolescents in India reported that on comparison with revised World Health Organization Quality-of-Life Scale, (WHOQOL-BREF), the PedsQL has poor to fair validity, concluding WHOQOL-BREF to be a better HRQoL instrument in the context [20]. It is also to be noted that the current study findings report better baseline HRQoL scores than the global healthy child norms, another factor of concern in relation to the validity of PedsQL in the context.

Over the past decade, a range of paediatric specific instruments have been developed in high-income settings, but literature on their validity and utility in low- and middle-income contexts is limited. Given the high burden of injuries in low- and middle-income countries, and the need for robust data on magnitude of injury burden, there is a need to revise tools that are culturally and contextually representative of the HRQoL measures in the population.

The major strengths of this study are good follow-up rates (87%) and cohort representation from secondary health facilities as well as a tertiary health facility. Our findings thus need to be cautiously interpreted with respect to the cohort characteristics, injury severity measures, reliability in parent reports for PedsQL, and the overall validity of HRQoL instruments in the context. 

Paediatric cohorts are unique, as children are growing, changing physically and psychologically; also, they differ in their interpretation and reporting of health outcomes from adults (proxy instruments) [21]. They are also likely to have better recovery outcomes as compared to adults. Vani et al. reporting on healthy child norms found higher summary scores by children (83.8) as compared to parent report (82.7) [22]. We used parent report of participant HRQoL and function to reduce potential for inconsistency within the age groups in the cohort. However, this is a potential source of bias. 

The study has a limitation in that that injury severity was not scored, and proxy measures such as days of hospitalisation and surgery were used to classify the severity of the injury. Over 70% of the cohort had a hospital stay of more than two days and less than two percent had a surgical intervention. Whilst unlikely, the immediate improvement in HRQoL scores can be driven by mild injury cases.

Although this study has demonstrated limited impact of injuries on HRQoL, our sample is hospital based where care seeking occurred following the injury event and may account for the rapid improvements observed. Health care seeking following a paediatric injury in India is not common practice; one study showed that in just over half of cases caregivers used home remedies rather than seek medical care for their injured child [23]. Future research should include community-based implementation of a validated tool in the Indian setting in order to examine outcomes for children where treatment was not sought for injuries, as it is likely they would be at greater risk of poorer outcomes.

## 5. Conclusions

The study findings report a high burden of hospitalization associated with falls and road traffic injuries in children aged 2–16 years. Proven interventions to prevent injuries from falls and road traffic in children are much needed in India. Government legislation on helmet wearing, seatbelts, and reducing speed around schools can make an impact [24]. Community-based programmes to improve home and workplace safety are also required [25,26]. 

Although this study did not find an impact at 12 months post-injury on functional outcomes and quality of life in children, there are currently few established rehabilitation programs for paediatric injuries in India, where rehabilitation is generally on a need basis [27]. Community-based rehabilitation for disabilities, outreach for spinal cord injuries has been demonstrated to have some success in India [28,29] and is an area where further research is required to develop and establish programs, and, in particular, human resources for rehabilitation, to ensure better outcomes and minimal long-term impact of injuries on quality of life and functioning into adulthood.

The findings confirm the need for development of context-specific tools, highlighting that the tools developed for high-income settings to measure health-related quality of life might not be directly transferable to low- and middle-income country settings. There is an urgent need for tested, validated instruments measuring health outcomes in children that can be recommended for standardized data collection for trauma registries, and for guiding early intervention and rehabilitation strategies. 

## Figures and Tables

**Table 1:** Baseline characteristics of children aged 2–16 years (*n* = 386).

Characteristic	*n* (%)
Age group	
2–4 years	105 (27.2)
5–7 years	82 (21.2)
8–12 years	103 (26.7)
13–16 years	96 (24.9)
Gender	
Male	276 (71.5)
Female	110 (28.5)
Education (*n* = 247) ^	
Not at school	53 (21.5)
Primary	108 (43.7)
Middle–Secondary	78 (34.8)
Any comorbidities	
Yes	6 (1.5)
No	380 (98.5)
Injury intent	
Unintentional	377 (97.7)
Intentional self-harm	1 (0.3)
Assault	8 (2.1)
Place of injury	
Home/residential institution	214 (55.4)
Street/highway	128 (33.2)
Other	44 (11.4)
Activity at time of injury	
Domestic work/personal hygiene/vital activities	302 (78.2)
Paid work	15 (3.9)
Other	69 (17.9)
Mechanism of injury	
Road traffic injury	138 (35.8)
Falls	187 (48.5)
Burn	22 (5.7)
Drowning	-
Poisoning	2 (0.5)
Mechanical	24 (6.2)
Others	13 (3.4)
* Severity	
Mild (LOS 0–1 day)	111 (30.1)
Moderate (LOS 2–7 days)	199 (53.9)
Severe (LOS 8+ days)	59 (16.0)
Surgery	
Surgery performed	7 (1.8)
No surgery	379 (98.2)
Cases referred	
Yes	296 (77.0%)
No	90 (23.0%)
Disposition at discharge	
Admitted	354 (91.7%)
Left against medical advice	1 (0.3%)
Died	15 (3.9%)
Unknown	16 (4.1%)

^ Includes children aged 6 years and older * 17 missing length of stay (LOS).

**Table 2:** Characteristics of road traffic injuries (*n* = 138).

Characteristic	*n* (%)
Mode of transportation	
Pedestrian	46 (33.3)
Bicycle	23 (16.7)
Two-wheeler	46 (33.3)
Car	7 (5.1)
Bus/truck	7 (5.1)
Other	8 (5.8)
Unknown	1(0.7)
Type of road	
Lane	5 (3.6)
Street	35 (25.3)
Main road	94 (68.1)
Unknown/others	4 (2.9)
Role of the injured	
Driver/operator	43 (31.2)
Passenger	65 (47.1)
Other	29 (21.0)
Unknown	1 (0.7)
Use of helmet if on two-wheeler (*n* = 46)	
Yes	5 (10.9)
No	40 (87.0)
Missing	1 (2.2)
Use of seatbelt if in car (*n* = 7)	
No	7 (1.8)
Person/object hit in the crash	
Vehicle	97 (70.3)
Road surface (pothole)	19 (13.8)
Other	22 (15.9)

**Table 3:** Characteristics of fall injuries (*n* = 187).

Characteristics of Fall Injuries	*n* (%)
Type of fall	
Fall from Height	
Electric Pole	8 (4.3)
Stairs/steps/ladder	28 (15.0)
Roof/building structure	69 (36.9)
Tree	15 (8.0)
Others	9 (4.8)
Fall from bed/chair/furniture	9 (4.8)
Fall on same level	
Slipping/tripping/stumbling	32 (17.1)
Other fall on same level from collision with, or pushing by, another person	7 (4.2)
Other	10 (5.0)
Reason for fall	
Slippery surface	93 (49.7)
Stumbling over object	24 (12.8)
Poor visibility	26 (13.9)
Dizziness	6 (3.2)
Others	35 (18.7)
Unspecified	3 (1.6)

**Table 4:** KOSCHI categories for children aged 2–16 years at baseline, 1, 2, 4, and 12 months after injury (*n* = 386).

Outcome Measure	Baseline	1 Month	2 Months	4 Months	12 Months
KOSCHI, *n*	*n* = 373	*n* = 282	*n* = 282	*n* = 277	*n* = 299
Severe disability *	-	2	2	0	0
Moderate disability *	1	12	6	3	0
Good recovery	1	45	15	15	0
Intact recovery	1	84	112	121	10

* Lower and upper combined due to small numbers.

**Table 5:** PedsQL by age group in children aged 2–16 years at baseline, 1, 2, 4, and 12 months after injury.

	Baseline		1 Month		2 Months		4 Months		12 Months	
	PedsQL Physical									
Age (years)	N	Mean (SD) *	N	Mean (SD) *	N	mean (SD) *	N	Mean (SD) *	N	Mean (SD) *
2–4	104	99.6 (2.5)	75	88.5 (27.2)	75	94.7 (19.0)	77	99.8 (1.4)	79	100 (0)
5–7	79	99.4 (3.3)	63	82.5 (32.4)	63	94.0 (17.4)	62	99.2 (6.4)	68	100 (0)
8–12	101	99.0 (4.6)	81	73.5 (33.7)	84	91.4 (23.4)	78	97.8 (12.9)	83	100 (0)
13–16	89	99.6 (2.7)	61	74.5 (31.7)	59	93.6 (15.6)	60	99.2 (6.5)	69	99.6 (3.4)
	**PedsQL Psychosocial**									
2–4	101	99.9 (0.9)	75	91.8 (21.1)	75	96.4 (14.6)	77	100 (0)	79	100 (0)
5–7	78	99.5 (3.0)	63	87.9 (24.1)	63	95.6 (13.3)	62	99.2 (6.4)	68	100 (0)
8–12	101	99.0 (3.9)	81	83.7 (26.2)	85	92.6 (21.2)	77	98.9 (6.4)	83	100 (0)
13–16	88	99.4 (3.8)	62	81.7 (23.6)	59	94.5 (12.9)	60	99.4 (4.3)	69	100 (0)

* standard deviation.
